# Ticks on game animals in the fragmented agricultural landscape of western Poland

**DOI:** 10.1007/s00436-021-07132-9

**Published:** 2021-03-31

**Authors:** Olaf Ciebiera, Andżelina Łopińska, Grzegorz Gabryś

**Affiliations:** 1 Department of Nature Conservation, Institute of Biological Sciences, University of Zielona Góra, Prof. Z. Szafrana 1, 65-516 Zielona Góra, Poland; 2 Department of Human Nutrition and Diet Therapy, University of Zielona Góra, Pałac Kalsk - Kalsk 67, 66-100 Sulechów, Poland; 3 Department of Zoology, Institute of Biological Sciences, University of Zielona Góra, Prof. Z. Szafrana 1, 65-516 Zielona Góra, Poland

**Keywords:** Ticks, Ixodida, red deer, roe deer, Eurasian wild boar, Poland, *Haemaphysalis concinna*

## Abstract

Ticks (Acari: Ixodida) are well known external parasites of game animals that cause serious veterinary and medical problems. The occurrence and geographical distribution of different species of ticks in Western Poland have changed over the last decades. The purpose of the present study was to determine the species spectrum and prevalence of ticks parasitizing three species of game animals, the Eurasian wild boar *Sus scrofa* L., red deer *Cervus elaphus* L., and roe deer *Capreolus capreolus* (L.) in two hunting districts in Lubuskie Province. In addition, the distribution of ticks on the host’s body and the intensity of infestation were determined. Ticks were collected from dead animals during the hunting seasons in 2013 and 2014, over the periods from May to June and from August to December. In total, 286 specimens were examined: 138 Eurasian wild boars, 8 red deers, and 140 roe deers. Altogether, 1891 ticks were collected. Three species of ticks were determined: *Ixodes ricinus* (L.), *Dermacentor reticulatus* (Fabricius, 1794), and *Haemaphysalis concinna* (C.L. Koch, 1844). *H. concinna* was recorded for the first time in Lubuskie Province.

## Introduction

Ticks are well-known vectors of pathogens causing diseases in humans and wildlife. In Poland, ticks are mostly responsible for tick-borne encephalitis (TBE) and Lyme borreliosis transmission, but cases of tularemia (*Francisella tularensis*), Q fever, spotted fever diseases, and other rickettsioses have also been listed as vectored by ticks (Kmieciak et al. 2016). The transmission of these diseases is associated with species of ticks that expand their range in Poland and other European countries, specifically *Dermacentor reticulatus* (Fabricius, 1794) and *Haemaphysalis concinna* (C.L. Koch, 1844). The enlargement of the geographic ranges in which ticks can survive may result in a higher number of people having contact with infected ticks. Most of the vector ticks have well-defined geographic distribution that has been determined by their adaptations to local abiotic environmental factors, such as relative humidity, temperature variation, micro environmental factors - soil moisture and soil permeability, and biotic factors, such as dense vegetation, humid leaf litter, and forests providing dense shade (Sonenshine 2018). Numerous recent reports suggest that climate change contributes to the ticks range expansion (Karbowiak 2014, Hvidsten et al. 2020, Medlock et al. 2013; Siroký et al. 2011).

In this context, considering the reports on the expanding distribution of ticks, we focused on the occurrence of ticks on wild game animals in Western Poland. The need for more information about the distribution and expansion of local tick species has been stressed by many authors (Adamska 2008; Doby et al. 1994). These aspects of tick ecology have widely been studied recently in reference to different groups of hosts, specifically reptiles (Nowak 2010), migratory birds (Ciebiera et al. 2019; Buczek et al. 2020), and mammals (Sawczuk et al. 2005; Adamska and Skotarczak 2007, Wodecka 2007, Michalik et al. 2012, Karbowiak et al. 2020). The knowledge of tick occurrence patterns in their natural habitats, their hosts and time of activity in different biotopes is important not only from the ecophysiographic point of view, but also shows global patterns of zoonoses emerging due to global warming. Additionally, the phenomenon of wild game animals, such as wild boar, roe deer, red deer, fox, and European badger migration into the cities or other human settlements becomes more frequent in recent years (Jakubiec and Jakubiec 2008). Moreover, humans exploit the same habitats for leisure and sport, at the same time exposing themselves to tick bites and tick-borne diseases. The game animals provide the essential link in pathogen life cycle (Wodecka 2007) and are the reservoirs of pathogens, such as *Borrelia burgdorferi* s. l., *Babesia microti*, *Babesia divergens, Anaplasma phagocytophilum,* and others (Stańczak et al. 2009; Sawczuk et al. 2005; Adamska and Skotarczak 2007; Michalik et al. 2012).

Depending on the life stage, ticks can choose different parts of the body for feeding. The larvae feed mainly on the legs of roe deer, adult ticks mainly on a neck, back and groin, and nymphs on ears and partially on the legs (Mysterud et al. 2014). The selection of feeding sites is important for co-feeding transmission of ticks-borne diseases, for example TBE and other pathogens (Buczek et al. 2006). In this context, we have examined the ticks distribution on hosts for better understanding potential consequences of co-feeding transmissions within one species and among different species of ticks.

To improve the tick control programmes, it is necessary to consider the hosts and the local distribution of ticks. The purpose of the present study was to determine tick species parasitizing on the most common game animals in Western Poland, specifically red deer, roe deer, and Eurasian wild boar and describe the distribution of the recorded tick species within the area of the host populations. The population of game animals as potential carriers of ticks has not been studied in Lubuskie Province before.

## Materials and Methods

### Study area

**Fig. 1:**
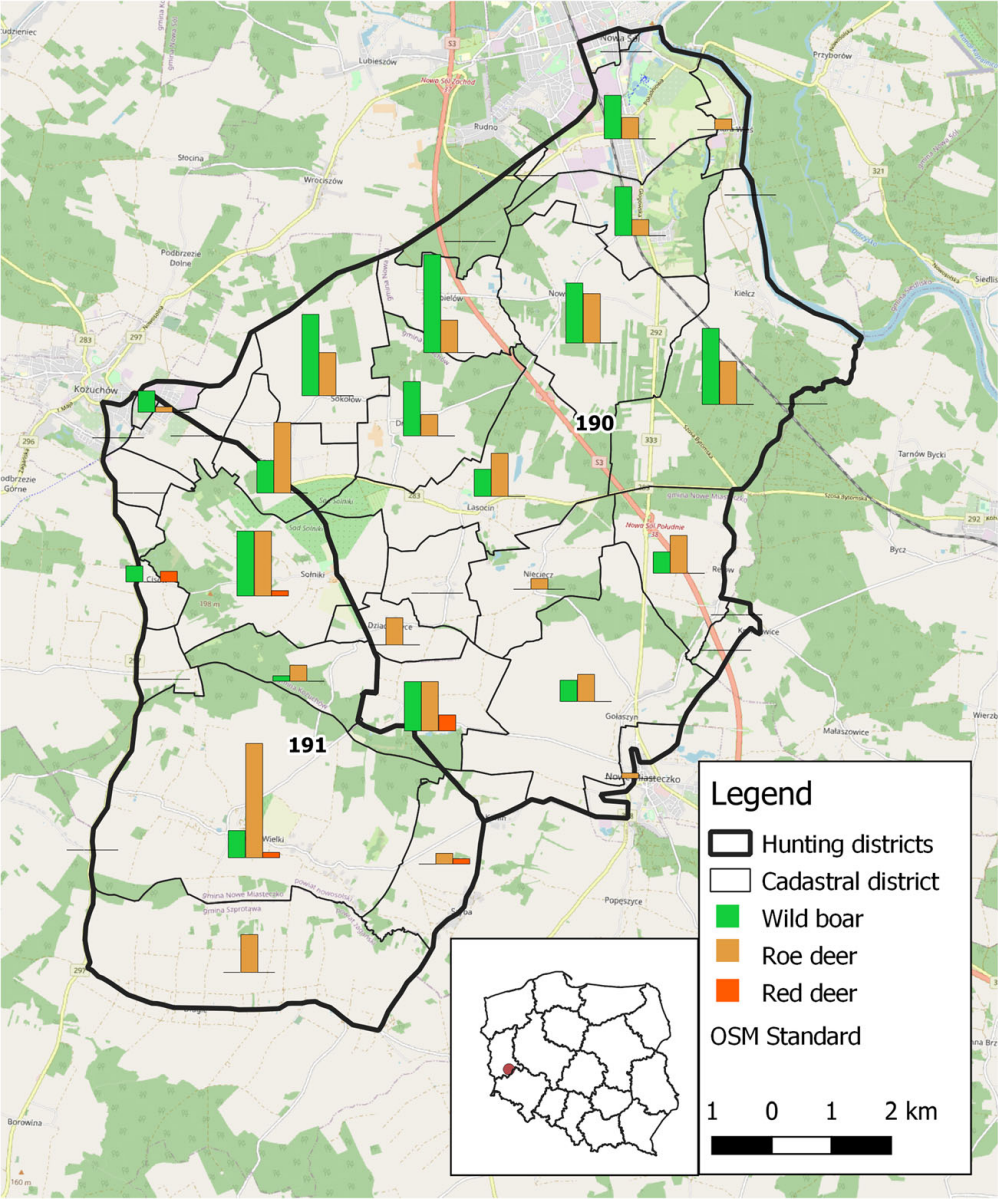
190 and 191 hunting districts localization in Lubuskie Province, Western Poland with spatial distribution of dead animals in cadastral districts. Map data copyrighted OpenStreetMap contributors (2020) and available from https://www.openstreetmap.org

The studies were carried out in two hunting districts (No. 191 and No. 190) in Lubuskie Province, Western Poland (Fig. 1). The proportions of level 2 land cover types were obtained from Corine Land Cover 2018 (copernicus.eu). The dominant habitats were arable lands (dominant 70.9% in district 191) and forests (dominant 24.5 % in district 190). The artificial areas covered about 7% and 5% in 190 and 191 districts, respectively. The total areas of the studied hunting districts were 7911.98 ha and 4474.63 ha for 191 and 190 hunting districts, respectively. The proportion of individual habitats in hunting districts was statistically highly significant (Chi^2^ with Yates = 361.92, df = 8, p <0.00001). The detailed habitat composition in hunting districts 190 and 191 is presented in table 1.

**Table 1:** Habitat composition [ha] in hunting districts 190 and 191 in Lubuskie State, Western Poland. The percentages of individual habitats in the hunting district are given in brackets

Hunting district	Urban fabric	Artificial, non-agricultural vegetated areas	Arable land	Permanent crops	Pastures	Heterogeneous agricultural areas	Scrub and/or herbaceous vegetation associations	Inland waters	Forests	Total:
190	463.2 (5.9%)	96.8 (1.2%)	4382.3 (55.4%)	117.4 (1.5%)	248.1 (3.1%)	588.9 (7.4%)	29.4 (0.4%)	45.7 (0.6%)	1940.2 (24.5%)	**7912.0**
191	158.6 (3.5%)	24.5 (0.5%)	3171.9 (70.9%)	79.6 (1.8%)	35.2 (0.8%)	211.7 (4.7%)	0.0 (0.0%)	0.0 (0.0%)	793.1 (17.7%)	**4474.6**
**Total:**	**621.9**	**121.3**	**7554.2**	**197.0**	**283.3**	**800.6**	**29.4**	**45.7**	**2733.3**	**12386.6**

In both examined hunting areas there dominate arable lands with oilseed rape, wheat, rye, barley, and maize, but fields with cabbage, sugar beet, and root vegetables are also relatively common. Buffer strips, which are refuges for animals in open areas, are common in this region. Second dominant habitat type is the broad leaved forest where animals feed and rest. The most common species in such forests are oaks *Quercus* spp., beech *Fagus sylvatica*, birch *Betula pendula*, and black locust *Robinia pseudoacacia*. The northern border of 190 district is formed by Oder river with a fragment of riparian forest and the town of Nowa Sól. The dominant coniferous species is Scots pine *Pinus sylvestris* in 191 district. The climate of the region under study is temperate, with precipitation of 500-600 mm/year and belongs to the hottest in the whole country of Poland. Annual average temperature is 9^o^C. Growing season encompasses about 235 days. Animal Husbandry Center (Ośrodek Hodowli Zwierzyny), where ticks were collected from dead animals, is placed in Nieciecz (Nowe Miasteczko community).

### Collection and identification of ticks

Ticks were collected from dead animals in 191 and 190 hunting districts in 2013 and 2014. The timing of tick collection depended on the hunting period, and covered the spring period from May to June and late summer-autumn period from August to December. The sites where animals were killed are presented in Figure 1, with reference to cadastral districts.

Dead animals were carefully inspected with protective gloves and ticks were removed from feeding sites and assigned to body regions: ears, head, neck, back, rump, groin, sternum and abdomen, legs. Ticks were collected up to 2-3 hours after the host’s death and no ticks leaving the hosts were observed during the collection period. All ticks were picked up directly from the host’s skin at the feeding site. Each tick was cleared and put individually in labelled tubes with 70% ethanol. The life cycle stage of each tick was determined. All specimens were examined and identified using the keys of Siuda (1993) and Estrada-Peña et al. (2004). For identification, binocular microscopes Nikon SMZ 800 and Nikon SMZ 3000 with software were used. Statistical analysis was performed using Statistica 10 Software and Excel 2010. Statistical test Chi-square was used according to Zar (1999). We also calculated the tick prevalence (i.e., the proportion of host individuals infested) and the intensity of infestation (i.e., the average number of ticks present on all infested individuals of a species) for each host species.

## Results and discussion

In total, 286 dead animals of three species were examined in 2013 and 2014, which were 8 red deers, 140 roe deers, and 138 Eurasian wild boars. Altogether, 1891 ticks were collected that belonged to three species: *Ixodes ricinus* (L.), *Dermacentor reticulatus* (Fabricius, 1794), and *Haemaphysalis concinna* (C.L. Koch, 1844) (Table2). The tick prevalence and intensity of tick infestation were determined for red deer (only 8 males inspected), roe deer and wild boar. All of red deer, 66% of roe deer and 49% of wild boar carried ticks (Table 2) and the mean intensity of infestation was 32.6 (min=4, max=87), 13.6 (min=1, max=103), and 1.5 (min=1, max=45) ticks per individual animal, respectively. For further analysis red deer were excluded because of the small number of individuals (only 8 males). No significant differences in prevalence (Chi^2^=0.2422, df=2, p=0.8859) and intensity of infestation (Chi^2^=3.3008, df=2, p=0.192) between hunting districts were found. The prevalence was higher in roe deer than in wild boar (Chi^2^=8.3762, p=0.0038, df=1). The proportion of infested females of Eurasian wild boar was significantly higher than males (Chi^2^=9.3414, p=0.0022, df=1), in contrast to roe deer, where the proportion of infested males was significantly higher than females (Chi^2^=12.766, p=0.0004, df=1).

**Table 2:** Distribution of ticks on hosts (A – number of examined hosts, a – number of infested hosts, M- male, F – female, L – larva, N – nymph)

Host (A/a)	Prevalence [%]	*I. ricinus*	*D. reticulatus*	*H. concinna*
		(M/F/L/N)	(M/F/L/N)	(M/F/L/N)
**Eurasian wild boar (138/67)**	**48.6**	**1/0/0/0**	**252/125/0/0**	
female (56/36)	64.3		164/70/0/0	
male (82/31)	37.8	1/0/0/0	88/55/0/0	
**Red deer (8/8)**	**100.0**	**25/59/0/0**	**163/14/0/0**	
male (8/8)	100.0	25/59/0/0	163/14/0/0	
**Roe dear (140/92)**	**65.7**	**335/865/3/30**	**3/5/0/0**	**4/7/0/0**
female (43/19)	44.2	7/23/0/0	3/4/0/0	
male (97/73)	75.3	328/842/3/30	0/1/0/0	4/7/0/0
**Total (286/167)**	**58.4**	**361/924/3/30**	**418/144/0/0**	**4/7/0/0**

In order to find temporal differences in prevalence and infestation intensity only 2 species (roe deer and wild boar) were chosen due to the fact that no red deer was available for study in the spring collection period. No significant differences in tick prevalence (Chi^2^=2.862, df=1, p=0.0933) in spring and autumn were found. However, the proportion of tick infestation of roe deer was significantly higher in spring than in autumn and infestation of wild boar was significantly higher in autumn than in spring (Chi^2^=12.759, df=1, p=0.0004). Although a difference was detected in the habitat structure for both hunting areas, the prevalence and infestation of ticks in individual game species did not differ. The structure of habitats in hunting districts does not affect these two indicators because animals can freely migrate between different types of habitats in the hunting districts and be exposed to ticks. At the same time, the killing of animals by hunters was not random, as the hunters shot the animals from pulpits placed permanently in the fields.

Feeding site selection was established for all examined individuals and was different for hosts species (Table 3). The occurrence of *H. concinna* is reported from Lubuskie Province, Western Poland, for the first time.

**Table 3:** Feeding site selection by ticks on hosts during study period (St. & abd. – sternum & abdomen), percentage in brackets (%)

Host	Ears	Head	Neck	Back	Rump	Groins	St. & abd.	Legs	Total
**Eurasian wild boar**	175 (46.3)	23 (6.1)	90 (23.8)	19 (5.0)	0 (0.0)	33 (8.7)	31 (8.2)	7 (1.9)	378
**Red deer**	14 (5.4)	0 (0.0)	180 (69.0)	10 (3.8)	1 (0.4)	46 (17.6)	10 (3.8)	0 (0.0)	261
**Roe deer**	57 (4.6)	37 (3.0)	723 (57.7)	5 (0.4)	16 (1.3)	391 (31.2)	21 (1.7)	2 (0.2)	1252

Table 2**.** Distribution of ticks on hosts (A – number of examined hosts, a – number of infested hosts, M- male, F – female, L – larva, N – nymph).

Table 3**.** Feeding site selection by ticks on hosts during study period (St. & abd. – sternum & abdomen), percentage in brackets (%).

Three species of ticks were found on game animals in Lubuskie Province: *I. ricinus*, *D. reticulatus,* and *H. concinna*. *I. ricinus* is widely distributed in Poland and is a known ectoparasite of game animals (Kadulski 1989; Dróżdż and Bogdaszewska 1997; Fryderyk 2000; Bogdaszewska 2005; Biaduń et al. 2007; Adamska 2008, Dwużnik et al. 2019, Karbowiak et al. 2020). Until recently, the distribution of *D. reticulatus* was limited to eastern Poland but many recent reports indicate that the range of this species has shifted to the West, what may be related to climate change and other factors (Nowak 2010; Kiewra and Czułowska 2013; Karbowiak 2014; Karbowiak et al. 2020). Until recently, the only known localities for *H. concinna* were in the vicinity of Troszyn village, West Pomerania Province, in north-western part of Poland. In XXI century, the reports on the occurrence of this species in other regions of Poland were published. The first record of *H. concinna* in Lower Silesia, SW Poland, was described by Kiewra et al. (2019) and from vegetation in the Greater Poland Province by Dwużnik et al. (2019). Our study reports the first record of *H. concinna* in Lubuskie Province, W Poland. 11 individuals (4 males and 7 females) feeding on three males of roe deer in 191 hunting district near Kożuchów, Szyba and Solniki were found in 2013. *H. concinna* is a known vector of tick-borne encephalitis virus (TBE), tularemia (*Francisella tularensis*), thrombocytopenia syndrome virus, *Rickettsia* spp., and *Babesia* spp. (Kiewra et al. 2019).

Game animals can be reservoirs or amplificators of tick borne diseases and are an essential link in the pathogens spread within and/or among hosts populations or support the pathogens’ vectors (i.e., the ticks) population in the ecosystem. The mean intensity of infestation for all species in the present study was 32.6 ticks per animal (min=4, max=87). The total prevalence of ticks on red deer in our study (100%) is very high in comparison to other studies. Research conducted in the whole territory of Poland in 1970 (Kadulski 1989) indicated the prevalence of *I. ricinus* at the level 50%, and 4% of D*. reticulatus*. In Poland at that time, *D. reticulatus* was only noted east of the Vistula river. Adamska (2008) indicated the prevalence of ticks in North-Western Poland at the level 44.2%, and found only one tick species, *I. ricinus*. The total prevalence of Spanish population of red deer *Cervus elaphus hispanicus* by *I. ricinus* amounted to 41.3% (Ruiz-Fons et al. 2006). In our study, all red deer were hunted during the period from September till December. In this period, males have a direct contact with one another within the flocks, what may be the essential factor for ticks spreading. Moreover, red deer show clear seasonal variation in the selection of the environment. In spring, they penetrate mainly meadows and reeds, while in summer and autumn they migrate to oak and beech forests, and in winter, their most important refuge are pine forests. Other reports also confirm the expanding range of *D. reticulatus* in western part of Poland (Nowak 2011; Karbowiak and Kiewra 2010; Kiewra and Czułowska 2013).

Over the past 35 years, the characteristic rise in average annual temperature is observed in Lubuskie Province, which results in the increase in frequency of hot days and the decrease in the number of days with frosts (Susek et al. 2018). Average annual temperature in 1981 was ca. 8.2^o^C, while in 2015 it was ca. 9.7^o^C. In southern part of Lubuskie Province, the number of days with frosts during the last 35 years was marked by a downward trend from ca. 42 in 1981 to ca. 29 in 2015 in Zielona Góra with an average precipitation at the level of 586 mm per year. The weather conditions may strongly promote the expansion of *D. reticulatus* and *H. concinna* to the new territories in Poland. Interestingly, we recorded the co-infestation by *D. reticulatus* and *I. ricinus* in mid-December on two individuals of red deer.

In our study, 66% of examined roe deer carried ticks which belonged to three species: *I. ricinus*, *D. reticulatus* and *H. concinna*. The mean intensity of infestation calculated for all studied individuals of all species was 13.6 (min=1, max=103). In studies carried out in 2004-2005, the tick prevalence in the roe deer population in north west Poland was at the level of 44.2% and the mean intensity of infestation was 3.6 (min=1, max=9) (Adamska et al. 2008). Moreover, only *I. ricinus* was found in that study (Adamska et al. 2008). Kadulski (1989) pointed the prevalence of *I. ricinus* at the level of 45%, with mean intensity of infestation 12.4, and *D. reticulatus* 1%, with mean intensity of infestation 2.0. The phenomenon of higher prevalence, higher mean intensity of infestation and the appearance of new species (Kiewra et al. 2019) clearly show the growth of *I. ricinus, D. reticulatus* and *H. concinna* populations in W Poland, as well as the potentially higher risk of spreading the tick borne diseases. However, mainly *I. ricinus* occurred and the participation of other species was very low on roe deer in our study, which proves that habitats of *I. ricinus* and its host intermingle.

Our study showed that the infestation intensity was significantly higher in roe deer males than in females. Similar results were obtained by Kadulski (1975). Roe deer males were several times more frequently attacked than roe deer females: 73% and 21%, respectively (Kadulski 1975). This phenomenon may be related to differences in their migration patterns. Males have larger individual territories, thus they penetrate the environment more strongly (Mysterud 1999). In consequence, the probability of encountering ticks by males may be higher. On the other hand, the study of Mysterud (1999) from southern Norway proves that males of roe deer are rather sedentary and do not take long migration distances until the time of severe winter conditions. Average migration distance was 3.8 km for males and 12.4 km for females (Mysterud 1999).

Feeding site selection pattern of ticks on roe deer was: neck > groins > ears > head > sternum&abdomen> rump > back > legs. Similar results were observed in Norway, where ears, neck and groins were also the most preferred body parts for attachment by ticks (Mysterud et al. 2014). The occurrence of ticks on the bodies of vertebrate animals is usually determined by the possibility of self-cleaning and the preferences in choosing the feeding site by individual stages of ticks (Kiffner et al. 2011). However, the stadial preferences were not considered in our study. Co-feeding or inter-stadial and species aggregation of ticks may enhance the transmission of tick borne-pathogens and lead to increased risk of infection (Kiffner et al. 2011).

Eurasian wild boar is responsible for the maintenance of *D. reticulatus* in the ecosystem. Our study shows that only one individual of *I. ricinus* was found on wild boar, whereas *D. reticulatus* was the superdominant. The total prevalence was 48.6%. Kadulski (1989) indicated the prevalence of *I. ricinus* at the level 16% and *D. reticulatus* 1%. In the study conducted by Adamska (2008), the wild boar carried only *I. ricinus* and its prevalence was only 6%. In Spain, the variation in species/subspecies was higher and 8 different ixodid ticks taxa were found: *Hyalomma marginatum marginatum* (68.7%), *Ripicephalus bursa* (14.6%), *Dermacentor marginatus* (9.3%), *Ripicephalus sanguineus* (2.5%), *Hyalomma lusitanicum* (1%), *Dermacentor reticulatus* (0.2%), *Ixodes ricinus* (0.1%) and *Hyalomma anatolicum excavatum* (0.05%) (Ruiz-Fons et al. 2006). The total prevalence in this study was 31% (Ruiz-Fons et al. 2006). The Eurasian wild boar is a reservoir of many viruses, bacteria, and parasites, including ticks that can infect domestic animals and humans (Meng et al. 2009). The distribution range and the population number of wild boars are increasing due to many factors, such as global climatic changes, availability of food resources, decreasing distance of escape (Bieber and Ruf 2006). African swine fever (ASF) caused by DNA virus of the Asfarviridae family is one of the major viral diseases in swine species and causes damage in domestic pig populations. African swine fever virus (ASFV) is transmitted to swine species during blood-feeding by soft ticks e.g. *Ornithodoros* spp. (Chae et al. 2017). Although there are no reports of ASF in hard ticks, the transmission of this disease should be investigated (Frant et al. 2017). Tick detection (especially soft-ticks) therefore, is essential in investigating the spreading of ASF in swine species (Chae et al. 2017). In our study, the females of the Eurasian wild boar were more frequently infested than the males. Kadulski (1989) reported exactly opposite results, where males were more infested than females. Eurasian wild boar lead rather sedentary way of life, migrations are rare, but the home range differs between European populations, e.g., in NE Germany it is 1185 ha, N Germany 316-470 ha, in Romania from 1060 to 12001 ha (Keuling et al. 2008, Jánoska et al. 2018). The causes of this behavior may be related to the differences in habitat preferences in different seasons, but this problem needs further investigation.

The spectrum of ticks species and the distribution patterns are diverse and depend not only on the nature of ecological opportunities in wild game animal’s habitats, but also on climatic factors. Similar conclusions can be drawn for other parasite-hosts relationships. The climate and habitat changes affecting the behaviour of birds, i.e., carriers of ticks and tick-borne pathogens, are accompanied by changes in the map of areas characterised by a high risk of tick-borne diseases in Europe (Buczek et al. 2020). The present study has potential implications for understanding the tick-borne disease transmission in the changing habitats with consideration of the expansion of tick species to new regions.
